# Exploiting fourth-generation synchrotron radiation for enzyme and photoreceptor characterization

**DOI:** 10.1107/S2052252524010868

**Published:** 2025-01-01

**Authors:** Tek Narsingh Malla, Srinivasan Muniyappan, David Menendez, Favour Ogukwe, Aleksandar N. Dale, Joseph D. Clayton, Dominique D. Weatherall, Prabin Karki, Shishir Dangi, Victoria Mandella, A. Andrew Pacheco, Emina A. Stojković, Samuel L. Rose, Julien Orlans, Shibom Basu, Daniele de Sanctis, Marius Schmidt

**Affiliations:** a Department of Physics University of Wisconsin-Milwaukee Milwaukee USA; b Department of Biology Northeastern Illinois University Chicago USA; c Department of Chemistry University of Wisconsin-Milwaukee Milwaukee USA; d European Synchrotron Radiation Facility (ESRF) Grenoble France; e European Molecular Biology Laboratory (EMBL) Grenoble France; UCL, United Kingdom

**Keywords:** structural biology, environmental chemistry, radiation damage, structure determination, macromolecular machines, time-resolved synchrotron serial crystallography

## Abstract

The upgrade of the European Synchrotron Radiation Facility (ESRF) in Grenoble, France to an Extremely Brilliant Source (EBS) is expected to enable time-resolved synchrotron serial crystallography (SSX) experiments with sub-millisecond time resolution. ID29 is a new beamline dedicated to SSX experiments at ESRF–EBS. Here, we report experiments emerging from the initial phase of user operation at ID29. We first used microcrystals of photoactive yellow protein as a model system to exploit the potential of microsecond pulses for SSX. Subsequently, we investigated microcrystals of cytochrome *c* nitrite reductase (ccNiR) with microsecond X-ray pulses. CcNiR is a decaheme protein that is ideal for the investigation of radiation damage at the various heme-iron sites. Finally, we performed a proof-of-concept subsecond time-resolved SSX experiment by photoactivating microcrystals of a myxobacterial phytochrome.

## Abstract 2

(Time-resolved) macromolecular crystallography at the new ID29 beamline at the ESRF is described.

## 1. Introduction

### 1.1. The ESRF–EBS ID29 beamline

X-ray crystallography, employed at synchrotron light sources, is a critical tool for determining the static structures of proteins at near-atomic resolution. Over 80% of all macromolecular structures deposited in the Protein Data Bank (Berman *et al.*, 2002 ▸) have been elucidated at cryogenic temperatures, which can compromise the biological relevance of the protein structures while still retaining susceptibility towards radiation damage (Garman & Weik, 2011 ▸, 2017 ▸; Suga *et al.*, 2015 ▸).

In the last decade, the introduction of X-ray free-electron lasers (XFELs) has transformed crystallography through the development of serial crystallography (SX; Chapman *et al.*, 2011 ▸; Boutet *et al.*, 2012 ▸; Redecke *et al.*, 2013 ▸; Kern *et al.*, 2012 ▸). SX allows data collection at physiological temperatures with minimal radiation damage (Lomb *et al.*, 2011 ▸; Chapman *et al.*, 2014 ▸; Nass *et al.*, 2015 ▸; Chapman, 2017 ▸; Mehrabi *et al.*, 2021 ▸) and also facilitates the initiation of reactions using an array of submicrometre-sized crystals. This capability makes SX an ideal choice for conducting time-resolved crystallo­graphy (TRX) studies, providing valuable insights into the dynamics of biological processes (Moffat, 1989 ▸, 2001 ▸; Schmidt, 2008 ▸, 2020 ▸, 2023*a* ▸; Pearson & Mehrabi, 2020 ▸).

The remarkable capabilities of XFELs have led to a surge in demand for SX applications. Therefore, it is highly desirable that accessible X-ray light sources are available at which the experiments can be conducted. SX experiments can now be conducted at highly brilliant third-generation and, recently, at fourth-generation synchrotron light sources with serial synchrotron crystallography (SSX), as the success of the SX technique at XFELs prompted a swift adaptation to these facilities (Schmidt, 2023*a* ▸; Gati *et al.*, 2014 ▸; Martin-Garcia, 2021 ▸; Stellato *et al.*, 2014 ▸; Beyerlein *et al.*, 2017 ▸; Nogly *et al.*, 2015 ▸; Meents *et al.*, 2017 ▸; Mehrabi *et al.*, 2021 ▸; Schulz *et al.*, 2018 ▸; Caramello & Royant, 2024 ▸; Mehrabi, Schulz, Agthe *et al.*, 2019 ▸; Mehrabi, Schulz, Dsouza *et al.*, 2019 ▸).

At fourth-generation synchrotron light sources, multi-bend achromat (MBA) lattices are utilized that substantially improve the emittance of the electron beam and, related to this, the brilliance of the X-ray beam. Concurrently, the integration of advanced detectors into the beamline enables highly sensitive and high-speed readout of the collected data. These collective upgrades help to enable serial crystallography at synchrotrons to offer serial and time-resolved crystallo­graphic experiments with microsecond-to-millisecond time resolution (Pearson & Mehrabi, 2020 ▸; Khusainov *et al.*, 2024 ▸), that can reach, with polychromatic X-ray sources, the pulse duration of 100 ps available at the synchrotron (Meents *et al.*, 2017 ▸; Martin-Garcia, 2021 ▸; Tolstikova *et al.*, 2019 ▸) as a practical alternative to serial femtosecond crystallography (SFX) at XFELs (Khusainov *et al.*, 2024 ▸).

The recent upgrade of the European Synchrotron Radiation Facility (ESRF) with a custom multi-bend achromat lattice to an ‘Extremely Brilliant Source’ (EBS; Raimondi *et al.*, 2023 ▸) resulted in a brilliance that is at least thirty times higher than before. As a result, a very intense X-ray beam can be focused without substantial loss of X-ray photons to spot sizes of the order of a few micrometres. These beams are exquisitely suited to interrogate microcrystals in SSX experiments. The new ID29 beamline that has been developed to take advantage of the ESRF–EBS is dedicated to room-temperature and time-resolved SSX (TR-SSX) experiments on biological macromolecules. The ID29 beamline can deliver of the order of >3 × 10^15^ hard X-ray photons (see Section 2.2 for details) with 1% bandwidth. However, there is a concern that a broader bandwidth impedes experiments with lower quality crystals that display substantial mosaic spread. Diffraction patterns are sensitive to crystals with larger mosaicities that result in elongated (streaky) Bragg reflections which are difficult to analyze. Serial Laue crystallo­graphy (SLX) on microcrystals has been applied at BioCARS at the Advanced Photon Source (APS), Argonne National Laboratory in Lemont, Illinois, which features a 5% bandwidth (Meents *et al.*, 2017 ▸; Martin-Garcia, 2021 ▸), and on beamline ID09 at the ESRF using a multilayer monochromator to reduce the bandwidth to 2.5% (Tolstikova *et al.*, 2019 ▸). Results suggest that serial crystallography with the Laue technique might be feasible at least with the high-quality, not too mosaic microcrystals interrogated during these experiments. Software developed for quasi-monochromatic data could be used for Laue data collected at BioCARS (Martin-Garcia, 2021 ▸) as well as on ID09 at ESRF (Tolstikova *et al.*, 2019 ▸). Apparently, averaging over a large number of observations can circumvent the need to determine the spectrum of the incident X-ray intensity (the so-called λ-curve) that is essential for merging Laue data (Ren & Moffat, 1995 ▸). One can expect that an X-ray bandwidth of 1% as implemented at ID29 will be beneficial for the successful collection of X-ray data from real-world crystals with large unit cells and elevated mosaicities.

### 1.2. Biological samples

Here, results from our early experiments at ID29 from February 2023 to September 2023 are reported that exploit the unique and special capabilities of this beamline. In these experiments, bacterial proteins with different functions were investigated. Microcrystals of photoactive yellow protein (PYP), a blue-light photoreceptor from *Halorhodospira halophila*, were used to explore the capability of microsecond X-ray pulses. A nitrite-reducing enzyme, cytochrome *c* nitrite reductase (ccNiR) from *Shewanella oneidensis*, was exploited to investigate potential secondary radiation damage, caused by 90 µs pulses, at heme centers. Finally, a first TR-SSX experiment was performed on the photosensory core module (PCM) of a bacterial phytochrome from the nonphotosynthetic myxobacterium *Stigmatella aurantiaca*.

PYP is a small protein (14 kDa, 125 amino acids) that covalently binds *p*-coumaric acid (pCA) to a conserved cysteine residue via a thioester bond. PYP (strain BN9626) forms highly diffracting crystals with various precipitants such as ammonium sulfate (Borgstahl *et al.*, 1995 ▸), sodium malonate (Tenboer *et al.*, 2014 ▸), polyethylene glycol (PEG) 2000 (van Aalten *et al.*, 2000 ▸; Anderson *et al.*, 2004 ▸) and PEG 4000 (Kort *et al.*, 2003 ▸). PYP from a different strain (SL-1) of *H. halophila* has also been crystallized using PEG 4000 (Anderson *et al.*, 2004 ▸). The photocycle of PYP has been extensively investigated by TRX (Ihee *et al.*, 2005 ▸; Jung *et al.*, 2013 ▸; Schotte *et al.*, 2012 ▸; Genick *et al.*, 1997 ▸) and time-resolved serial femto­second crystallography (TR-SFX; Tenboer *et al.*, 2014 ▸; Pande *et al.*, 2016 ▸; Hosseinizadeh *et al.*, 2021 ▸; Pandey *et al.*, 2020 ▸).

CcNiR is a decaheme enzyme that catalyzes the six-electron reduction of nitrite to ammonia. It is an important enzyme in the nitrogen cycle and is essential in agriculture for ammonia generation from nitrate fertilizers. X-ray dose effects are expected to be detected at the ten heme iron sites when ccNiR is exposed to X-ray radiation. The structure of ccNiR has previously been solved at room temperature using Laue crystallo­graphy (Youngblut *et al.*, 2012 ▸) at BioCARS and at cryogenic temperature using a home source (Ali *et al.*, 2019 ▸). Serial crystallography is particularly important for the investigation of ccNiR. CcNiR has been featured as an example to motivate mix-and-inject experiments (Schmidt, 2013 ▸), in which substrate is mixed with microcrystals and, after a delay, injected into the X-ray interaction region. In this way, an enzymatically catalyzed reaction is triggered by diffusion and its progress is probed by an X-ray pulse (Schmidt, 2013 ▸, 2020 ▸; Stagno *et al.*, 2017 ▸; Kupitz *et al.*, 2017 ▸; Olmos *et al.*, 2018 ▸; Dasgupta *et al.*, 2019 ▸; Malla *et al.*, 2023 ▸; Ishigami *et al.*, 2019 ▸; Mehrabi, Schulz, Agthe *et al.*, 2019 ▸; Pandey *et al.*, 2021 ▸). With sub-millisecond time resolution, mix-and-inject experiments with ccNiR will become feasible, enabling observation of the reduction of nitrite in real time.

Phytochromes are red/far-red light receptor enzymes that were originally discovered in plants, with homologs in bacteria and fungi. They exist in photosynthetic and nonphotosynthetic bacteria, with a diversity of functions from the synthesis of light-harvesting complexes and carotenoids to the control of cell motility (Auldridge & Forest, 2011 ▸). Myxobacteria are distinctive among prokaryotes due to the presence of a multicellular stage in their life cycle known as fruiting bodies. In *Stigmatella aurantiaca*, the formation of fruiting bodies is controlled by red and far-red light, implicating bacterio­phyto­chrome (BphP) signaling (Woitowich *et al.*, 2018 ▸; Qualls *et al.*, 1978 ▸; White *et al.*, 1980 ▸). BphPs share a typical domain architecture comprising three conserved domains (PAS–GAF–PHY) known as the photosensory core module (PCM) and a C-terminal effector domain with enzymatic activity, usually a histidine kinase (HisK). BphPs sense red/far-red light and display a reversible photocycle triggered by the *Z*-to-*E* isomerization of a central chromophore, biliverdin (BV). This isomerization results in the formation of distinct states called Pr and Pfr (red and far-red light-absorbing states, respectively). The effector domain perceives structural changes caused by light absorption through a distance of about 110 Å. Well defined structural changes characterize the photocycle of this protein, involving the rearrangement of the water network in the BV-binding pocket and conformational changes of highly conserved regions of the protein that stabilize the BV chromophore. Most notable is the conformational change of a stretch of amino-acid residues called the sensory tongue that transitions from a β-strand in the Pr state to a an α-helical conformation in the Pfr state (Takala *et al.*, 2014 ▸; Burgie *et al.*, 2016 ▸). The conserved pyrrole water (PW) that stabilizes rings A to C of the BV chromophore is photo-ejected as early as a few picoseconds after photo-initiation in the absence of the sensory tongue, as revealed by TR-SFX experiments (Claesson *et al.*, 2020 ▸). Crystal structures of BphPs in the Pfr state determined with sufficient resolution to resolve water molecules (Burgie *et al.*, 2016 ▸; Yang *et al.*, 2011 ▸) point to the PW returning back to the BV-binding pocket in the Pfr state. Full-length phytochromes are challenging to crystallize, but the PCM, lacking the effector domain, forms crystals that diffract to 2 Å resolution and beyond (Sanchez *et al.*, 2019 ▸; Burgie *et al.*, 2014 ▸; Woitowich *et al.*, 2018 ▸; Essen *et al.*, 2008 ▸). *S. aurantiaca* harbors two BphPs, SaBphP1 and SaBphP2, that bind BV and share similar domain compositions. Most recently, cryo-EM structures of the full-length and truncated PCM of SaBphP2 in the distinct red and far-red light-absorbing states have been determined (Malla *et al.*, 2024 ▸). For the first time, a phytochrome heterodimer with individual subunits in distinct conformations was characterized when the protein was exposed to white light. While it has been hypothesized in the past that phytochrome heterodimerization may occur and be functionally relevant, direct structural evidence was missing until these cryo-EM structures of SaBphP2 were published. Recent TR-SFX experiments on SaBphP2-PCM microcrystals have been conducted at the Spring-8 Angstrom Compact X-ray Laser (SACLA) facility in Japan. BV isomerization was trigged by red-light pulses, and the resulting structures were probed 5 ns and 33 ms after light illumination (Carrillo *et al.*, 2021 ▸). Significant structural displacements of the covalently bound bilin chromophore (*Z*-to-*E* isomerization of the BV chromophore) were observed that trigger a bifurcated signaling pathway that extends through the entire protein. However, the structural transition to the Pfr state is characterized by large domain reorientations (Malla *et al.*, 2024 ▸) and the transition of the sensory tongue to an α-helix (Takala *et al.*, 2014 ▸; Burgie *et al.*, 2016 ▸). None of these events were observed even at 33 ms. To investigate longer time scales, the new ID29 beamline at ESRF is equipped with a tape drive that enables a method for the collection of structural information on longer-lived intermediates of SaBphP2-PCM.

## 2. Methods

### 2.1. Sample preparation

PYP was overexpressed in *Escherichia coli* and purified as reported previously (Kort *et al.*, 1996 ▸; Tenboer *et al.*, 2014 ▸; Borgstahl *et al.*, 1995 ▸). The protein was concentrated to 100 mg ml^−1^ in a low-salt protein buffer consisting of 10 m*M* 4-(2-hydroxyethyl)piperazine-1-ethanesulfonic acid (HEPES) and 50 m*M* sodium chloride (NaCl) at pH 7.5. PYP microcrystals, denoted PYP_Mal_, were grown using sodium malonate as precipitant. Details are reported elsewhere (Tenboer *et al.*, 2014 ▸). PYP microcrystals with PEG as precipitant (PYP_PEG_) were grown using a mix-and-stir method with a precipitant solution consisting of 100 m*M* 2-(*N*-morpholino)ethanesulfonic acid (MES) and 40%(*w*/*v*) polyethylene glycol (PEG) 4000 at pH 6.5. Briefly, 0.2 ml concentrated PYP was transferred into a glass vial and 0.8 ml of the precipitant described above was slowly added dropwise (1:4 protein:precipitant ratio) with vigorous stirring. The suspension was stirred for ∼4 h and incubated for a further 24 h at room temperature. PYP microcrystals with average dimensions of approximately 20 µm appeared within 24 h. The crystal density was determined using a Neubauer cell-counting chamber, revealing a density of 4 × 10^8^ crystals per millilitre.

The ccNiR protein was overexpressed in the *S. oneidensis* TSP-C strain and purified in accordance with previously reported methods (Youngblut *et al.*, 2012 ▸; Ali *et al.*, 2019 ▸). CcNiR microcrystals were prepared using the following conditions:

*Condition 1.* The protein was concentrated to 80 mg ml^−1^ in a buffer consisting of 150 m*M* NaCl, 50 m*M* HEPES, 30 m*M* ammonium sulfate at pH 7. A precipitant solution was prepared consisting of 40% PEG 4K, 200 m*M* sodium malonate, 100 m*M* MES buffer at pH 6.5. The crystals were prepared by mixing the protein solution and the precipitant solution in a 2:1 ratio. Crystal-like particles grew within a week. Despite appearing visually to be crystalline, the particles did not diffract [Supplementary Fig. S1(*a*)].

*Condition 2.* The protein was concentrated to 40 mg ml^−1^ in 20 m*M* HEPES buffer at pH 7. The precipitant solution was 28% PEG 4K, 100 m*M* triethanolamine at pH 7.5. The crystals were prepared by mixing equal amounts of protein solution and precipitant. The mixture was stirred for approximately 4 h and was subsequently incubated for an additional two days at room temperature. The resulting microcrystal suspension contained a large amount of unusable amorphous particles in addition to diffracting microcrystals [Supplementary Fig. S1(*b*)].

*Condition 2-1.* The crystalline particles from condition 1 were collected by mild centrifugation and dissolved in 20 m*M* HEPES buffer at pH 7. They were finally recrystallized using condition 2 [Supplementary Fig. S1(*c*)]. The resulting microcrystals were thin plates and were heterogenous in size [Supplementary Fig. S1(*c*)]. Most crystals were about 20 µm in edge length [Supplementary Fig. S1(*c*)]. The microcrystals were concentrated to approximately 4 × 10^6^ crystals per millilitre for data collection.

The PCM of *S. aurantica* bacteriophytochrome 2 (SaBphP2-PCM) was overexpressed and purified as reported previously (Carrillo *et al.*, 2021 ▸). Large crystals of SaBphP2-PCM with dimensions of 100 × 100 × 100 µm were grown by the hanging-drop vapor-diffusion method with a protein concentration of 30 mg ml^−1^, 425 µl of 0.17 *M* ammonium acetate, 0.085 *M* sodium citrate tribasic dihydrate pH 5.6, 25.5%(*w*/*v*) PEG 4000, 15%(*v*/*v*) glycerol (cryo-screen solution) and 75 µl 20%(*w*/*v*) benzamidine hydrochloride (an additive) under dark conditions at 16°C. For the preparation of microcrystals, 60 mg ml^−1^ protein was mixed in batch mode with the same precipitant as described above in a 2:3 protein:precipitant ratio. The mixture was seeded with a few microlitres of a slurry obtained by finely crushing a large crystal with a glass bead (Hampton Research). The mixture was incubated for four days in the dark at 16°C. The SaBphP2 microcrystals were thin plates and, in contrast to the ccNiR microcrystals, were quite uniform in size, approximately 25 µm across [Supplementary Fig. S1(*d*)]. The microcrystals were collected and concentrated to ∼10^9^ crystals per milllilitre.

### 2.2. Data collection

Data were collected on beamline ID29 at the ESRF. The beamline was operating at a 231.25 Hz X-ray repetition rate at an energy of 11.56 keV with ∼1% (116 eV) bandwidth. The microcrystals were exposed to an X-ray pulse of 90 µs duration containing ∼10^11^ photons. The beam was focused to a spot size of 4 × 2 µm at the sample. Diffraction patterns were recorded using a JUNGFRAU 4M detector (Leonarski *et al.*, 2018 ▸) placed 150 mm away from the sample (2 Å at the edges, 1.8 Å in the corners).

**Figure 1:**
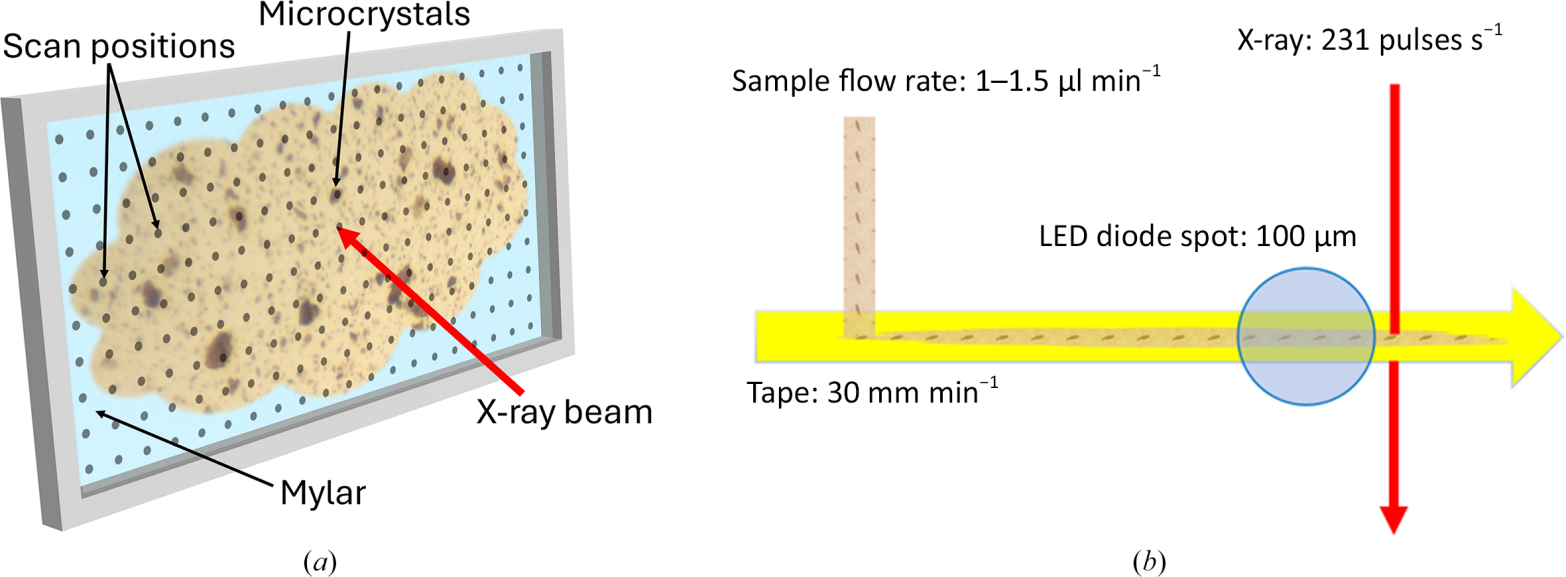
Two approaches for sample delivery at ESRF beamline ID29. (*a*) Fixed target. The microcrystal slurry is sandwiched between two Mylar films sealed by a metal mount and aligned in front of the X-ray beam. The chip is raster-scanned serially at pre-defined positions as shown by black dots. (*b*) Schematic depiction of the tape-drive setup. The microcrystal slurry is applied to a tape drive which transports the sample to the X-ray beam. Light from a laser or from a light-emitting diode (LED) can be used to initiate light-driven reactions in photoactive proteins before the crystals reach the X-ray interaction region.

Complete data sets for PYP, ccNiR and SaBphP2 were collected using sheet-on-sheet (SOS) chips [Fig. 1 ▸(*a*)] (Doak *et al.*, 2024 ▸). 3–5 µl of crystal slurry was sandwiched between two Mylar films (13 µm thickness) and sealed inside a metal mount. The portion of the chip containing the microcrystals was aligned in front of the X-ray beam. The chip was then continuously scanned with synchronized X-ray pulses with a step size of 20 µm horizontally as well as vertically. Data collection from each chip took about 8 min using this method, resulting in 81 800 detector readouts.

TR-SSX data for SaBphP2-PCM [Fig. 1 ▸(*b*)] were collected with a tape-drive device (Beyerlein *et al.*, 2017 ▸). The crystal slurry was applied at a rate of 1–3 µl min^−1^. By moving at a speed of 30 mm min^−1^, the tape delivers the microcrystals into the X-ray beam. Immediately before the X-ray beam, the microcrystals were illuminated for 200 ms with red light (λ = 640 nm) from a light-emitting diode (LED) that continuously illuminated the crystal slurry. The LED light was focused to a spot size of 100 µm. The total LED light energy density experienced by a microcrystal was 12 mJ mm^−2^. The time required by the crystals to travel from the center of the LED focal spot to the X-ray interaction region determines the time delay. With this setup, a 700 ms time-resolved data set was recorded. A dark data set (without LED illumination) was collected from PYP microcrystals using the tape drive to validate the feasibility of this data-collection method.

### 2.3. Data processing and analysis

Data collection was monitored and pre-processed in real time using the in-house *nplive* and *autoCryst* programs (Coquelle *et al.*, 2015 ▸), which also provided general feedback on spatial resolution and hit rate. All data were re-processed with the *CrystFEL* (version 0.10.1) suite of programs (White, 2019 ▸; White *et al.*, 2016 ▸). *Indexamajig* was used to index and integrate the diffraction patterns. The detector geometry and distance were refined by *geoptimiser* (Yefanov *et al.*, 2015 ▸) using high-quality data collected from PYP samples (see below). Indexing ambiguities (when necessary) were resolved using the *ambigator* program. Merging and scaling of the intensities were performed with *partialator*. Figures of merit and other data statistics were calculated using *compare_hkl*and *check_hkl*. The full intensities were converted to structure-factor amplitudes using software based on the *CCP*4 suite of programs (Agirre *et al.*, 2023 ▸). Exemplary shell scripts can be found in the supplementary material to Pandey *et al.* (2020 ▸).

For all data sets, the structures were refined with *Phenix* (Liebschner *et al.*, 2019 ▸), with manual inspection and adjustments performed in *Coot* (Emsley *et al.*, 2010 ▸). Statistics on data processing and refinement are given in Table 1 ▸. The structural comparison and the generation of all protein-display figures were performed in *UCSF ChimeraX* (Pettersen *et al.*, 2021 ▸). Difference electron-density (DED) maps were (if applicable) calculated from data collected after light excitation and reference data collected in the dark as described previously (Schmidt, 2023*b* ▸; Pandey *et al.*, 2020 ▸).

## 3. Results and discussion

### 3.1. Serial synchrotron crystallography (SSX) on beamline ID29 with PYP

We used both a fixed target and a tape drive to collect SSX data from PYP microcrystals on beamline ID29. The data-collection statistics are shown in Table 1 ▸. The PYP_PEG_ crystals diffracted to higher resolution than the PYP_Mal_ crystals. The PYP_PEG_ crystals belonged to space group *P*6_5_. The previously published structure with PDB code 4wla was used as a search model for molecular replacement. Using the X-ray data obtained with the fixed target and tape drive, structures were solved to resolutions of 1.8 and 1.9 Å, respectively (Table 1 ▸).

The PYP_PEG_ crystals appeared to be superior compared with the PYP_Mal_ crystals. The PYP_PEG_ microcrystals have sharper edges and visually appear more crystal-like. However, it is challenging for gas dynamic nozzles (GDVNs; DePonte *et al.*, 2008 ▸) to flow high-viscosity PEG solutions. Therefore, the PYP_Mal_ crystals were used in previous XFEL experiments (Tenboer *et al.*, 2014 ▸; Pande *et al.*, 2016 ▸; Pandey *et al.*, 2020 ▸). In fact, the XFEL experiments, conducted with GDVNs, might have been impossible with the PYP_PEG_ crystals. Microcrystals suspended in viscous PEG are best used with fixed targets and tape drives, both of which were used in this study.

**Figure 2:**
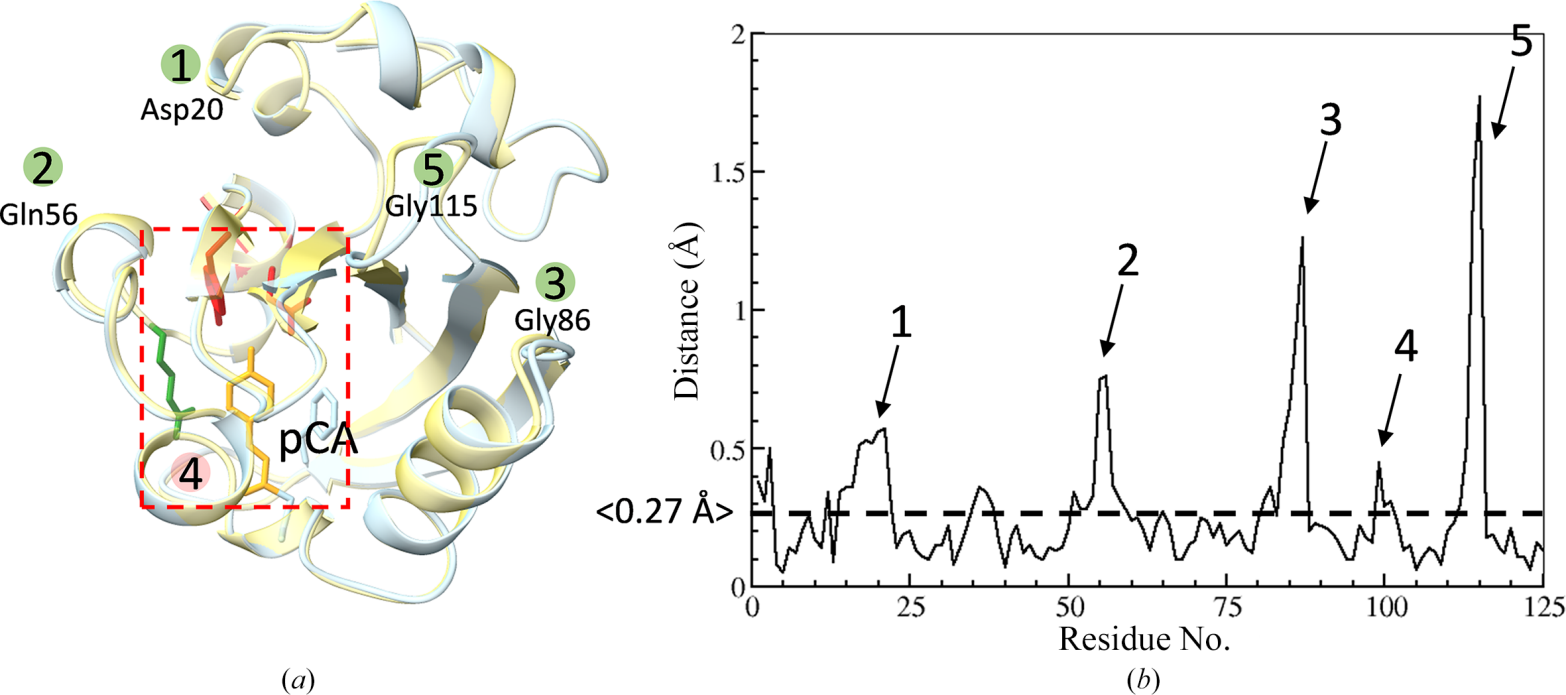
Structure of PYP. (*a*) The structure of PYP (blue) in space group *P*6_5_ determined by fixed-target SSX compared with that in space group *P*6_3_ (yellow) determined at the LCLS by SFX (PDB entry 4wla, reference structure). The pCA chromophore pocket is marked by the red box. Various residues are shown. The locations of four of the five distance peaks marked in (*b*) are denoted with green circles together with the representative residues. The pale red circle denotes peak 4 in (*b*) where there are no structural differences. (*b*) Distances between equivalent C^α^ positions in the superposed PYP structures. The average deviation is 0.27 Å. Peaks refer to specific locations marked in (*a*). There are no significant structural changes for peak 4, but this peak is very pronounced in the low-temperature comparison from van Aalten *et al.* (2000 ▸). Distances and the overlay were determined using *lx_lsqman* (Kleywegt & Jones, 1994 ▸).

Structures from PYP crystals grown in PEG show differences compared with those grown from malonate [Figs. 2 ▸(*a*) and 2 ▸(*b*)]. The differences are essentially in accordance with a previous study (van Aalten *et al.*, 2000 ▸), except for peak 4 in Fig. 2 ▸, which is absent in our comparison and very pronounced in the study by van Aalten and coworkers. Van Alten and coworkers speculated that the change of space group caused a significant reduction in intermolecular contacts between the symmetry-related molecules and might also account for the conformational heterogeneity (van Aalten *et al.*, 2000 ▸). Crystal contact analysis of our structures in *ChimeraX* revealed that the *P*6_3_ crystal form is the denser of the two, in accordance with van Aalten *et al.* (2000 ▸). PYP in space group *P*6_5_ only forms four contacts between symmetry-related copies within 5 Å, while in space group *P*6_3_ eight contacts are formed. It should be emphasized that our structures were both obtained at room temperature, while the structures of van Aalten and coworkers were obtained at cryogenic temperature, which might have an additional effect, in particular near amino acid Met100 (peak 4). Despite the denser packing of the *P*6_3_ crystals, the crystals grown from PEG diffract to better resolution. Based on previous experiences with the malonate-grown crystals at BioCARS (unpublished work), we suspect that these crystals feature a larger mosaicity, which results in diffraction to lower resolution.

The highly diffracting PYP_PEG_ crystals were used for calibration and detector geometry refinement and for optimizing the key parameters for data collection from other proteins.

### 3.2. Structural characterization of ccNIR with room-temperature SSX

**Figure 3:**
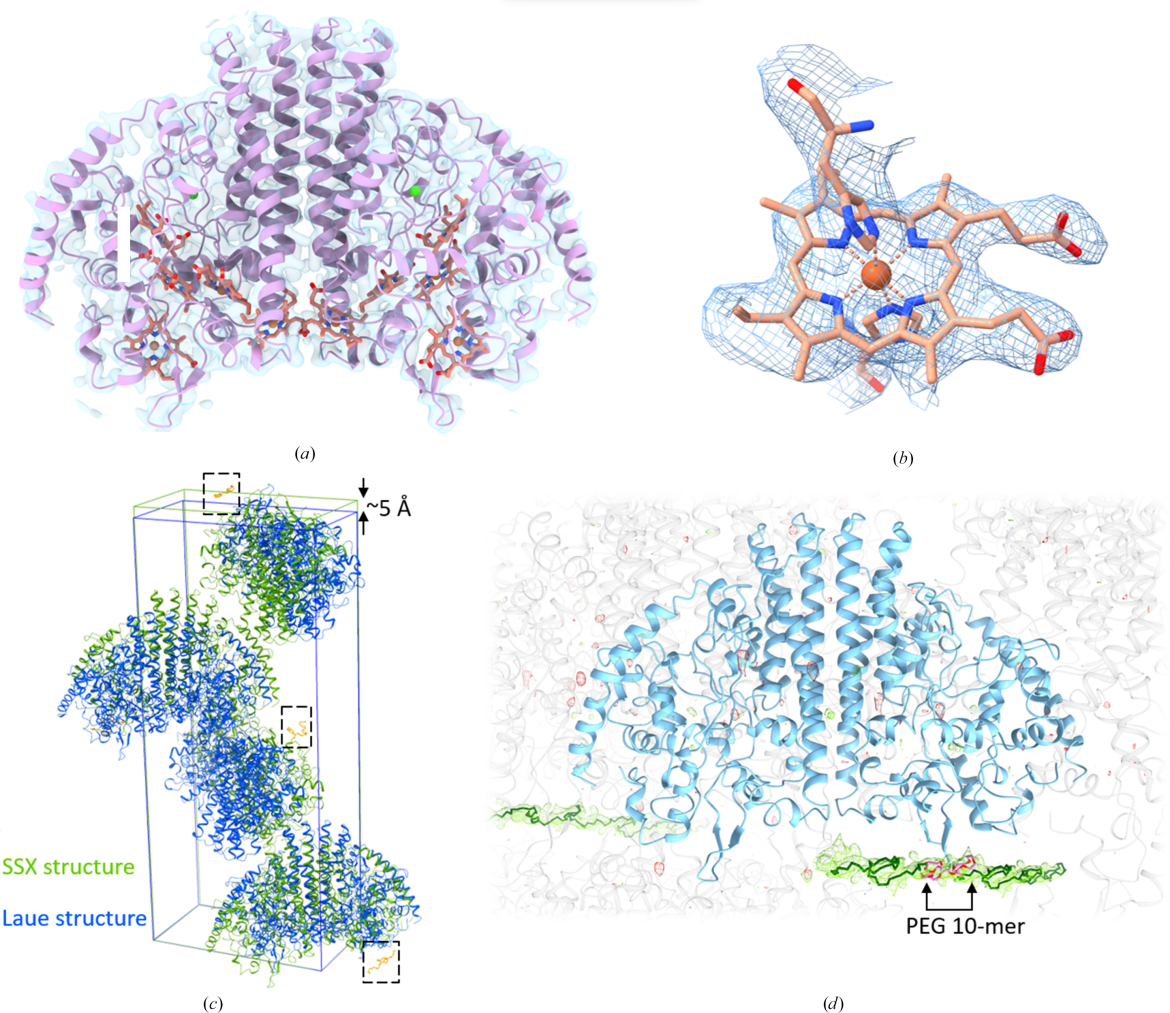
Structure of ccNiR. (*a*) The room-temperature structure is shown in light pink and the electron-density map is shown in transparent light blue. The ten heme cofactors are shown in salmon and the Ca ions are shown in bright green. (*b*) Close-up view of one of the heme cofactors. The heme(s) as well as the side chains in (*a*) are well resolved even at a resolution of 3.3 Å. (*c*) Comparison of the SSX structure (green) with the previously published Laue structure (blue). The unit cell of the SSX structure is larger by 5 Å in one of the axes. Another difference is the presence of a PEG molecule in the SSX structure, which is marked by dotted squares. (*d*) Close-up view of the asymmetric unit of ccNiR. The omit difference map is shown at ±3σ. The PEG molecule traverses the entire width of the unit cell. Only ten PEG oxyethylene monomers can be modeled in the asymmetric unit, while the rest are generated by crystallographic symmetry.

With the ccNiR microcrystals a full data set with 3106 indexed diffraction patterns was obtained, resulting in the first room-temperature structure [Fig. 3 ▸(*a*)] of ccNIR determined by SSX. CcNiR crystallized in space group *P*2_1_2_1_2_1_ with a dimer in the asymmetric unit. This is similar to the previously published (room-temperature) Laue structure (Youngblut *et al.*, 2012 ▸) and another structure collected at cryogenic temperature (Ali *et al.*, 2019 ▸). The third axis, *c*, however, is longer by 5 Å (228.2 Å versus 223 Å) compared with the previous structures [Fig. 3 ▸(*c*), Table 1 ▸]. The packing of the ccNiR molecules within the unit cell is also different [Fig. 3 ▸(*c*)]. Due to this disparity, molecular replacement was required to determine the phases. The Laue structure (PDB entry 3ubr) was used as a search model. A single solution was identified and refined.

Despite the limited resolution (3.3 Å), the side chains, including the heme cofactors, are clearly resolved [Figs. 3 ▸(*a*) and 3 ▸(*b*)]. Upon refinement, a strong positive difference density appears next to one of the protomers [Figs. 3 ▸(*c*) and 3 ▸(*d*)]. This density traverses parallelly through the width of the unit cell (axis *b*). It is likely that the PEG 4000 used for crystallization weaves around the ccNiR molecules and is potentially involved in stabilizing the crystal lattice.

It is unlikely that the presence of PEG hinders the activity of ccNiR. The PEG is situated far away from the opening of the channels that guide ions, electrons and water into the active heme coordination sites. A thorough analysis requires time-resolved mix-and-inject experiments (Schmidt, 2013 ▸) to pinpoint the actual path taken by the substrate.

### 3.3. Pump–probe TRX with SaBphP2

**Figure 4:**
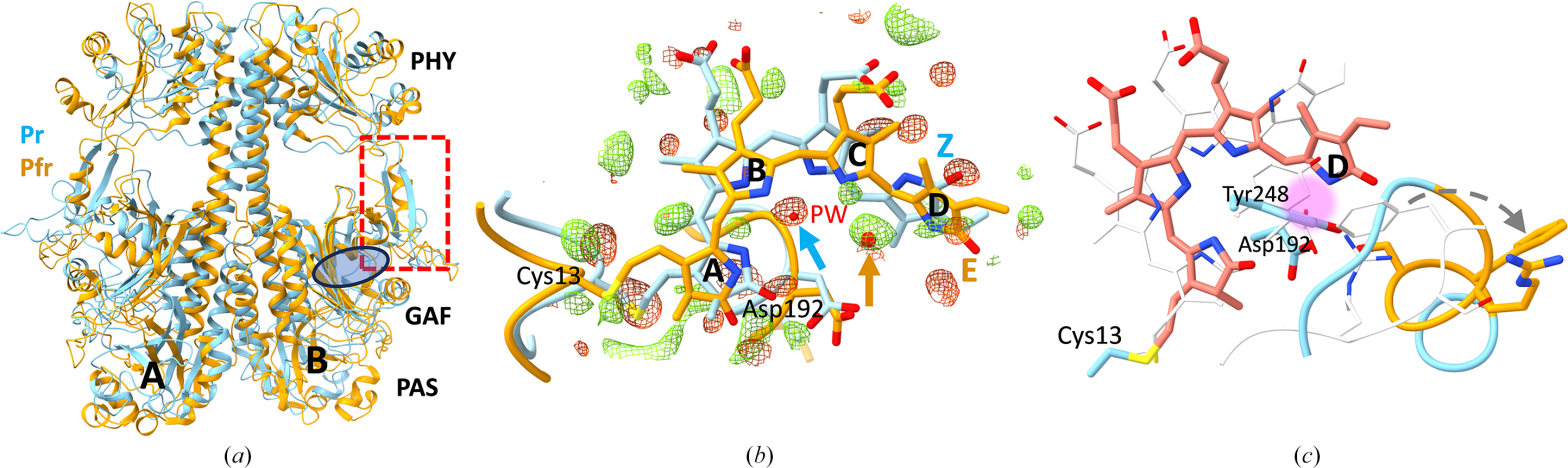
SaBphP2. (*a*) SaBphP2 in the Pr (blue, this work) and Pfr (orange) states. The structure of the Pfr state was determined by single-particle cryo-EM (PDB entry 8upm). Subunits *A* and *B* of the homodimer as well as the PAS, GAF and PHY domains are marked. The position of the chromophore pocket is marked by the blue oval in subunit *B*. The sensory tongue (red dotted square) transitions from a β-sheet in Pr to an α-helical conformation in Pfr. Note: the sensory-tongue transition is not observed in our experiments even 700 ms after photoabsorption. (*b*) DED features (green, positive; red, negative) around BV 700 ms after photoactivation. The four rings of BV (A–D) are labeled. The structure of the chromophore with ring D in the *E* configuration as found at a 33 ms pump–probe time delay (PDB entry 7jri) is overlayed (orange) as a guide to the eye. The conserved pyrrole water (PW) is ejected from its initial position (blue arrow) and returns at 700 ms at a new site (orange arrow). (*c*) Overlay of BV in the Pr (this work) and Pfr [salmon; PDB entry 8upm as in (*a*)] states. The sensory-tongue structure in the Pfr state is shown in blue and selected residues in the PR*X*SF motif are highlighted in orange. A hydrophilic core area near the returning PW location is marked by the purple cloud

For the SaBphP2 crystals, 83 000 indexed patterns were obtained from the chips as a reference (dark) data set (Table 1 ▸). Similarly, 36 000 patterns were obtained from the pump–probe (700 ms) experiment using the tape drive. A reference model was refined against the dark data (Table 1 ▸) using the SaBphP2 room-temperature structure with PDB entry 6ptq as the initial model. A weighted DED map was calculated from the pump–probe TR-SSX data and the reference data as described in detail previously (Schmidt, 2019 ▸, 2023*b* ▸). The DED map showed the presence of difference electron density at 700 ms [Fig. 4 ▸(*b*)]. The signal is weaker in this data set than in previous experiments conducted at SACLA (Carrillo *et al.*, 2021 ▸), despite the higher total red-light energy used to excite the reaction (5 mJ mm^−2^ at SACLA versus 12 mJ mm^−2^ applied here). The deterioration of the signal might have to do with the different modes of data collection (fixed target for dark and tape for the 700 ms data). Clearly, more experiments are necessary to examine this behavior. Nevertheless, the signal is distinguishable from the noise and makes chemical sense. The DED features, localized to the BV chromophore, indicate displacement of the BV and its moieties. For illustrative purposes, a chromophore structure with ring D in the *E* configuration determined 33 ms after reaction initiation (Carrillo *et al.*, 2021 ▸; PDB entry 5c5k) is overlayed on the reference structure determined here [Fig. 4 ▸(*b*)]. The structure explains many of the positive DED peaks. The strong negative DED feature at the position of one of the waters, known as the pyrrole water (PW), reaffirms earlier observations of the same PW, which was also absent at the earlier time points of 5 ns and 33 ms determined at SACLA (Carrillo *et al.*, 2021 ▸). At 700 ms, the photo-ejected PW has not returned back to its original position. Instead, a positive signal below the D ring indicates a putative relocation site [Figs. 4 ▸(*b*) and 4 ▸(*c*)]. The returning PW is attracted by a hydrophilic environment [Fig. 4 ▸(*c*)] that might be important for the transition to the Pfr state, including the formation of an α-helical sensory tongue.

Although not observed here, the transformation of the β-stranded sensory tongue to an α-helical conformation [Fig. 4 ▸(*a*)] is essential for photosignal transfer from the BV chromophore to the PHY domain. The helical transformation is likely to begin with displacements of residues in the (conserved) PR*X*SF motif, which is part of the sensory tongue [Fig. 4 ▸(*a*)] and is located near the chromophore [Fig. 4 ▸(*c*)]. The side chain of Arg457, which initially forms a salt bridge with Asp192 in the Pr state, turns away in the Pfr state [Fig. 4 ▸(*c*)]. This has been observed in crystal structures and cryo-EM structures of pure Pfr states (Burgie *et al.*, 2016 ▸; Takala *et al.*, 2014 ▸; Wahlgren *et al.*, 2022 ▸; Malla *et al.*, 2024 ▸). Concurrently, the side chain of Ser459, which was initially oriented outwards in the Pr state, rotates inwards and forms a hydrogen bond to Asp192 in the Pfr state [Fig. 4 ▸(*c*)]. However, the precise molecular mechanism of this action is not well understood, necessitating further investigation. Since Arg–Asp salt bridges are generally stronger and longer-lasting than Ser–Asp hydrogen bonds, an additional trigger mechanism is required to break this connection.

The roles of key residues in the PR*X*SF motifs have been individually studied through point mutations (Anders *et al.*, 2013 ▸; Zhang *et al.*, 2013 ▸). Most notably, an unusual near-red-shifted RpBphP3 from the photosynthetic bacterium *Rhodo­pseudomonas palustris* has a threonine instead of a proline in the PR*X*SF motif. It is the only phytochrome identified to date with such a variation in this critical motif. With a single point mutation placing proline back instead of threonine in the PR*X*SF motif, RpBphP3 becomes a classical red/far-red light-absorbing phytochrome (Yang *et al.*, 2015 ▸). Arg457 has been identified as crucial for stabilizing the Pr state, with its mutation impeding the thermal reversion from the Pfr state to the Pr state (Zhang *et al.*, 2013 ▸). Similarly, Ser459 is crucial for stabilizing the Pfr state and its mutation prevents the formation of Pfr. On the other hand, bathy phytochromes exist that are thermally stable in the Pfr state. With a mutation of the serine in the PR*X*SF motif to an alanine, the bathy phytochrome becomes a classical Pr- to Pfr-state phytochrome with a thermally stable Pr state (Anders *et al.*, 2013 ▸). Interestingly, mutation of the phenylalanine prevents Pfr formation, although this residue is not directly involved in stabilization of the Pfr state (Anders *et al.*, 2013 ▸). The importance of the sensory-tongue residues for the function of phytochromes warrants additional structural investigations of SaBphP2 mutants.

Our difference map at 700 ms provides additional insight. Fig. 4 ▸(*c*) illustrates the chromophore-binding pocket in the Pr and Pfr states observed by crystallography (Burgie *et al.*, 2016 ▸) and recently obtained by cryo-EM (Malla *et al.*, 2024 ▸). In the Pr state, Phe460 is buried close to the hydrophobic environment of ring D, stabilizing the Pr state. In the Pfr state, the polar side groups of Tyr248 and Asp192, and the nitrogen of the ring D pyrrole, form a hydrophilic site near the returning PW location [Fig. 4 ▸(*c*)]. We hypothesize that the robust hydrophilicity of the assembly may expel the hydrophobic side chain of Phe460 out of the chromophore pocket, which serves as the additional trigger mechanism mentioned above. The ensuing rotation of Phe460 potentially serves as a plausible catalyst for initiating the forward α-helical transformation process and the initiation of further conformational changes of the entire protein scaffold, as recently observed by cryo-EM (Malla *et al.*, 2024 ▸).

## 4. Conclusion and outlook

Our data show that TR-SSX data can be collected at ID29 with high spatial resolution. For PYP and the phytochrome, excellent data were collected that rival the quality of data collected from the same proteins at XFELs (Tenboer *et al.*, 2014 ▸; Sanchez *et al.*, 2019 ▸; Carrillo *et al.*, 2021 ▸). The data quality of ccNiR, with an *R*_split_ of 36% (Table 1 ▸), could have been improved further by collecting more diffraction patterns. At the time, we were limited by the amount of ccNiR sample available to us. In our opinion, in particular with respect to accumulating data for a high-resolution reference structure, it is better to collect high-multiplicity serial data which might be contaminated by some radiation damage than to collect a low-multiplicity data set with large *R*_split_ values. Accordingly, the same fixed target can be slightly displaced and the scan repeated, or large scanning steps can be replaced with smaller steps to increase the available number of detector readouts, even when some of the crystals are interrogated twice or even multiple times. In our experiment, the scanning steps were conservatively chosen to be 20 µm apart to prevent any contamination from the previous X-ray exposure. Decreasing the scanning step by 10 µm would have increased the number of diffraction patterns by a factor of four. A lower *R*_split_ value and a better resolution would likely be the result.

The uptake of an electron by ccNiR creates a current that can be used by sensors or exploited as an (exotic) energy source. CcNiR can be used as an amperometric electrode for the detection or measurement of unwanted chemical species (Strehlitz *et al.*, 1996 ▸). Accurately quantifying nitrite concentrations has a direct application in monitoring nitrite in drinking water and wastewater-treatment facilities. Such a device would require an electron donor to function. Therefore, understanding the electron pathways of these reduction processes could also spark the design of novel artificial electron donors.

The successful room-temperature structure determination of ccNiR opens avenues for future time-resolved mix-and-inject serial crystallography (MISC) experiments (Schmidt, 2013 ▸). MISC enables the visualization of the complete reaction cycle in order to better understand enzymatic mechanisms (Schmidt, 2013 ▸, 2020 ▸; Stagno *et al.*, 2017 ▸; Kupitz *et al.*, 2017 ▸; Olmos *et al.*, 2018 ▸; Dasgupta *et al.*, 2019 ▸; Malla *et al.*, 2023 ▸; Ishigami *et al.*, 2019 ▸; Mehrabi, Schulz, Agthe *et al.*, 2019 ▸; Pandey *et al.*, 2021 ▸) and capture intermediates that populate the reaction pathway. The reaction in ccNiR microcrystals can be triggered by mixing with a nitrite solution. With a proper electron donor such as methyl viologen (Schmidt, 2013 ▸), structural changes should occur from the binding of nitrite to the generation of the final product, ammonium. At room temperatures, the *Shewanella* ccNiR turnover time is 1.2 ms (Youngblut *et al.*, 2012 ▸). Small molecules such as nitrite diffuse efficiently into the crystals. Assuming a diffusion coefficient of 2 × 10^−5^ cm^2^ s^−1^ and plate-like 10 × 10 × 2 µm crystals (Supplementary Fig. S1), we anticipate diffusion times around 200 µs (Schmidt, 2013 ▸), which match the 90 µs X-ray exposures well. Meaningful MISC experiments with ccNiR are likely to be feasible. The observation window should be increased by (i) maximizing the nitrite concentration to quicklyreach a stoichiometric substrate concentration (Schmidt, 2020 ▸) in the unit cells and (ii) decreasing the temperature (Schmidt *et al.*, 2013 ▸) as an attempt to slow down the ccNiR turnover rate.

Concerns exist about X-ray radiation damage inflicted by the large number of X-ray photons during a single 90 µs pulse (Garman & Weik, 2011 ▸). At XFELs the diffraction-before-destruction principle (Neutze *et al.*, 2000 ▸; Chapman *et al.*, 2006 ▸, 2014 ▸; Nass *et al.*, 2015 ▸) is the reason that structures are determined essentially free of (visible) radiation damage. It is a matter of intense and systematic study whether the X-ray pulses used at ID29 inflict substantial radiation damage, the disorder resulting from which would diminish the number of X-ray photons diffracted into Bragg reflections, in particular at high resolution. By comparing the electron density of an iron-containing protein (myoglobin) determined at an XFEL with that determined by SSX at the PETRA-III synchrotron at the Deutsches Elektronen Synchrotron (DESY) in Hamburg, radiation damage was observed at the iron site in the SSX data but not in the SFX data (Mehrabi *et al.*, 2021 ▸). However, the SSX X-ray exposures that were used at PETRA-III were about 37 ms long, in contrast to those at ID29, which are about three orders of magnitude shorter. Very weak, if any, tell-tale DED is detectable near any of the ten heme irons in ccNiR *F*_o_ − *F*_c_ difference maps. In the light of the limited amount of data collected and the limited resolution, further experiments are required. Because of the many heme irons in the ccNiR that are easily photoreduced, ccNiR crystals are ideal for these investigations.

The pump–probe experiment on SaBphP2-PCM is particularly noteworthy. While XFELs have been used for pump–probe experiments with this phytochrome on various time scales, including milliseconds (Claesson *et al.*, 2020 ▸; Carrillo *et al.*, 2021 ▸), XFELS are mainly used to study ultrafast processes. Longer time scales are not prioritized, although longer-lived intermediates are present in phytochromes whose structures need to be determined (Carrillo *et al.*, 2021 ▸). Pump–probe experiments on very long time scales are also feasible at XFELs using tape drives (Kern *et al.*, 2018 ▸) and potentially using fixed targets with the hit-and-return technique (Schulz *et al.*, 2018 ▸). It needs to be established whether the quasi-monochromatic, single 10–40 fs X-ray pulses available at XFELs bear an advantage in terms of resolution and radiation damage compared with the 90 µs exposures used here. Another method that can determine macromolecular structures at near-atomic resolution is single-particle cryo-EM (Cheng, 2015 ▸; Nakane *et al.*, 2020 ▸; Yip *et al.*, 2020 ▸). TR-cryo-EM (Frank, 2017 ▸; Dandey *et al.*, 2020 ▸; Lorenz, 2024 ▸) is currently being developed. However, TR-cryo-EM is still in its early stages and may not be sufficient for detecting subtle conformational changes. As shown here, an essential knowledge gap left by both TR-SFX and cryo-EM can be filled in. The large number of synchrotrons worldwide and their completed or emerging upgrades (Caramello & Royant, 2024 ▸) are poised to open up room-temperature TR-SSX with a few-microsecond time resolution to a wider scientific community.

## Supplementary Material

PDB reference: cytochrome *c* nitrite reductase, SSX structure, 9cuf

PDB reference: photoactive yellow protein, room-temperature structure, 9d10

PDB reference: *Stigmatella aurantiaca* bacteriophytochrome photosensory core module, 9d2h

Supplementary Fig. S1. DOI: 10.1107/S2052252524010868/ro5043sup1.pdf

## Figures and Tables

**Table 1:** Data-collection and refinement statistics Values in parentheses are for the highest resolution shell.

	PYP_PEG_	PYP_PEG_	PYP_Mal_†	Phytochrome (dark)	Phytochrome (700 ms)	CcNiR
Data collection						
Method	Fixed target	Tape drive	Fixed target	Fixed target	Tape drive	Fixed target
Space group	*P*6_5_	*P*6_5_	*P*6_3_	*P*2_1_	*P*2_1_	*P*2_1_2_1_2_1_
*a*, *b*, *c* (Å)	41.36, 41.36, 118.87	41.36, 41.36, 118.87	66.9, 66.9, 40.8	83.69, 83.40, 86.87	83.69, 83.40, 86.87	50.84, 95.47, 228.20
α, β, γ (°)	90, 90, 120	90, 90, 120	90, 90, 120	90, 90, 107.63	90, 90, 107.63	90, 90, 90
Total No. of reflections	2377266	2482326	137796	9667624	13702833	291409
No. of hits	36668	44954	71585	252933	51132	28464
No. indexed	37078‡	31027	2412	83498	36049	3106
Resolution range (Å)	118.87–1.80 (1.85–1.80)	118.87–1.90 (1.95–1.90)	57.94–2.50 (2.57–2.50)	119.04–1.80 (1.85–1.80)	83.40–2.50 (2.57–2.50)	228.18–3.33 (3.39–3.33)
Unique reflections	10896 (513)	9124 (448)	3708 (183)	69862 (732)	39628 (1979)	17548 (860)
Multiplicity	134.89 (42.75)	209.91 (47.1)	37.16 (25.1)	486.74 (313.8)	345.79 (241.8)	19.37 (12.3)
*R*_split_ (%)	10.68 (305.88)	8.95 (225.16)	34.21 (104.27)	9.65 (23.79)	13.53 (217.11)	36.57 (240.50)
Completeness (%)	100 (100)	100 (100)	100 (100)	89.55 (69.32)	100 (100)	100 (100)
CC_1/2_	0.99 (0.65)	0.99 (0.22)	0.77 (0.23)	0.99 (0.98)	0.99 (0.20)	0.95 (0.18)
CC*	0.99 (0.33)	0.99 (0.61)	0.93 (0.61)	0.99 (0.99)	0.99 (0.58)	0.99 (0.55)
Refinement						
Reflections used	9689	9039	Not refined	50696 (to 2.1 Å)§		16704
*R*_cryst_/*R*_free_	0.15/0.19	0.18/0.20		0.19/0.26		0.23/0.28
R.m.s.d., bond lengths (Å)	0.002	0.003		0.01		0.004
R.m.s.d., bond angles (°)	0.632	0.715		1.25		0.941
No. of waters	305	32		383		

† Calibration specimen: the data are sufficient for initial beamline calibration, but not for refinement.

‡ Multi-lattice indexing was turned on, which is why there are more indexed diffraction patterns than hits.

§ Refinement was conducted to 2.1 Å resolution, which is lower than the high-resolution limit of the data due to an irregular Wilson plot beyond this resolution.
