# Supplementary material for: Exploiting fourth-generation synchrotron radiation for enzyme and photoreceptor characterization
Source: IUCrJ. 2025 Jan 1;12(Pt 1):36–48. doi: 10.1107/S2052252524010868 (PMC11707700; doi:10.1107/S2052252524010868)
Supplement: Supplementary file 1 [file m-12-00036-sup1.pdf]

IUCrJ

Volume 12 (2025)

Supporting information for article:

Exploiting fourth-generation synchrotron radiation for enzyme and photoreceptor characterization

Tek Narsingh Malla, Srinivasan Muniyappan, David Menendez, Favour Ogukwe, Aleksandar N. Dale, Joseph D. Clayton, Dominique D. Weatherall, Prabin Karki, Shishir Dangi, Victoria Mandella, A. Andrew Pacheco, Emina A. Stojković, Samuel L. Rose, Julien Orlans, Shibom Basu, Daniele De Sanctis and Marius Schmidt

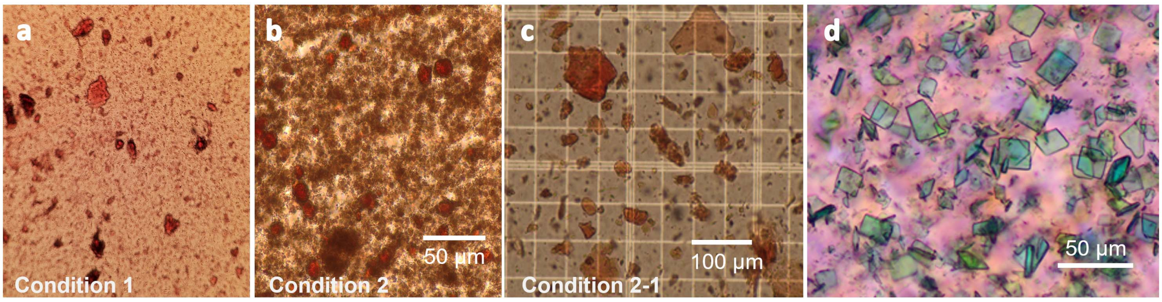

Figure S1 CcNiR and SaBphP2-PCM crystals. (a-c) ccNiR crystals: (a) Crystals grown in condition 1. Crystals did not diffract. (b) Crystals grown in condition 2. The crystal slurry contains amorphous particles in addition to well diffracting crystals. (c) The condition 2-1 is essentially re-crystallization of condition 1 aggregates with condition 2 resulting in improved, well diffracting crystals. (d) Typical sample of SaBphP2-PCM crystals.
